# Supplementary material for: Facile Transformation from Rofecoxib to a New Near-Infrared Lipid Droplet Fluorescent Probe and Its Investigations on AIE Property, Solvatochromism and Mechanochromism
Source: Molecules. 2023 Feb 15;28(4):1814. doi: 10.3390/molecules28041814 (PMC9967153; doi:10.3390/molecules28041814)
Supplement: Supplementary file 1 [file molecules-28-01814-s001.zip › molecules-2171205-supplementary.pdf]

Supporting Information

Facile Transformation from Rofecoxib to a New Near-infrared Lipid Droplet Fluorescent Probe and its Investigations on AIE Property, Solvatochromism. and Mechanochromism.

Yongbo Wei[1, 2], Wei Liu[1], Zexin Wang[1, 2], Nannan Chen[1], Jingming Zhou[1], Tong Wu[1], Yuqiu Ye[1], Yanbing Ke[1], Hong Jiang[1, *], Xin Zhai[2, *] and Lijun Xie[1, *]

[1] Fujian Provincial Key Laboratory of Screening for Novel Microbial Proucts, Fujian Institute of Microbiology, Fuzhou, Fujian 350007, P.R. China

[2] Key Laboratory of Structure-based Drug Design and Discovery, Ministry of Education, School of Pharmaceutical Engineering, Shenyang Pharmaceutical University. Shenyang, Liaoning 110016, P. R. China

*** Correspondence:**

Hong Jiang

E-mail: Jianghong709@163.com

Xin Zhai ORCID: 0000-0002-5585-1959

E-mail: zhaixin_syphu@126.com

Lijun Xie ORCID: 0000-0002-6503-6587

E-mail: lijunxie8224@outlook.com

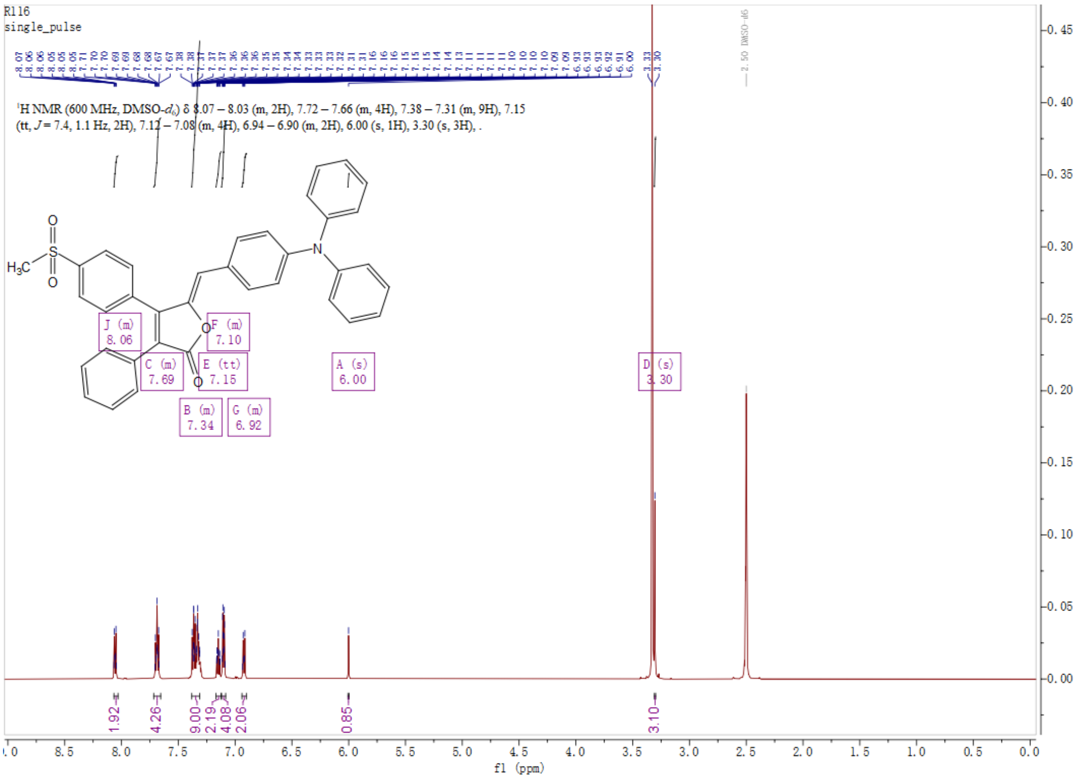

Figure S1. ^{1}H NMR spectrum of **BY1** in DMSO-d_6.

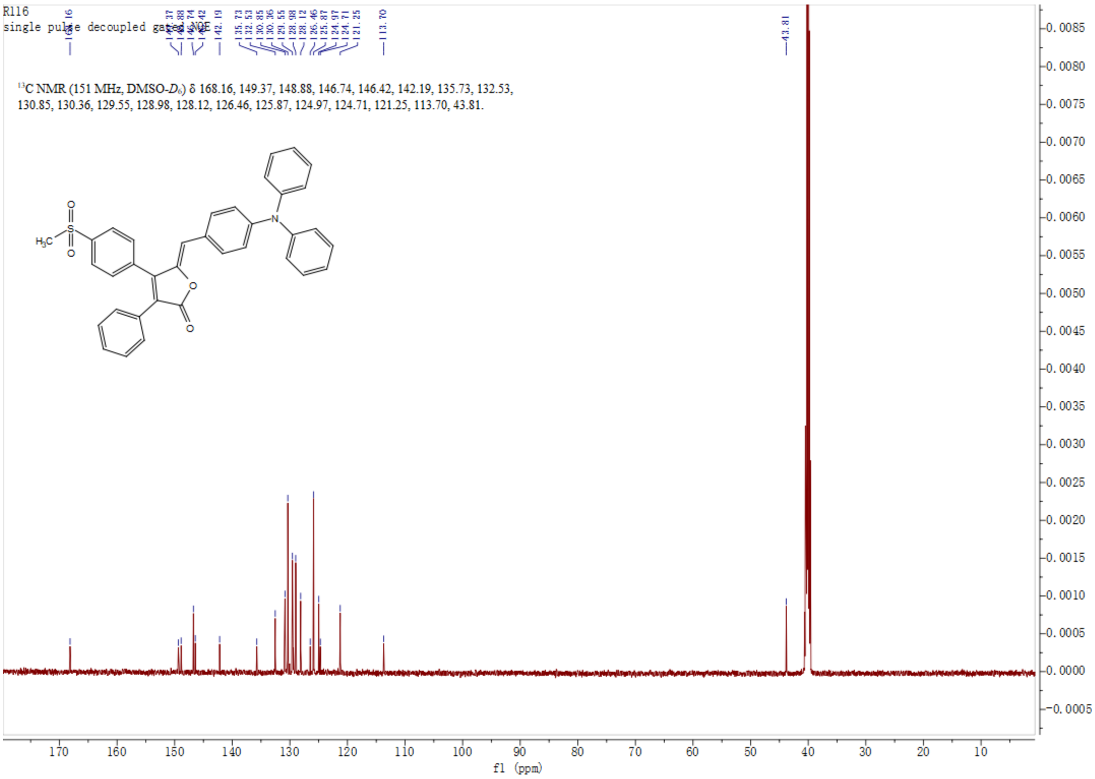

Figure S2. ^{13}C NMR spectrum of **BY1** in DMSO-d_6.

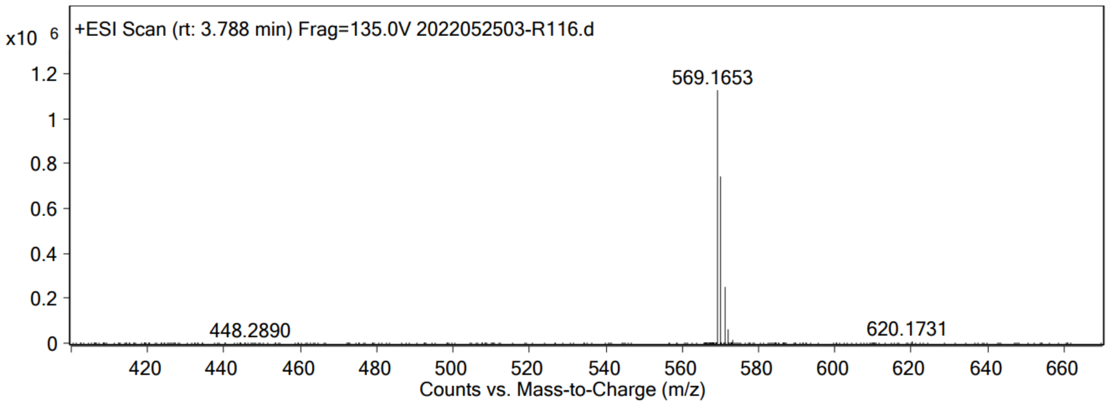

Figure S3. HRMS spectrum of **BY1**

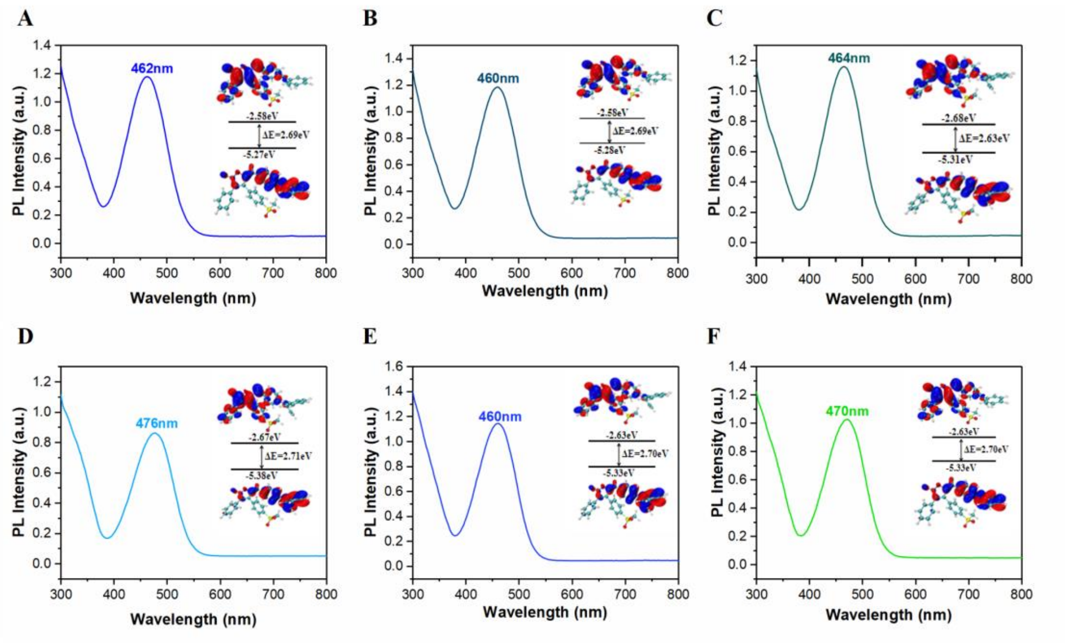

Figure S4. The absorption spectra of **BY1** in DMSO (A), DMF (B), EtOH (C), TCM (D), THF (E), DCM (F).(50 μM).

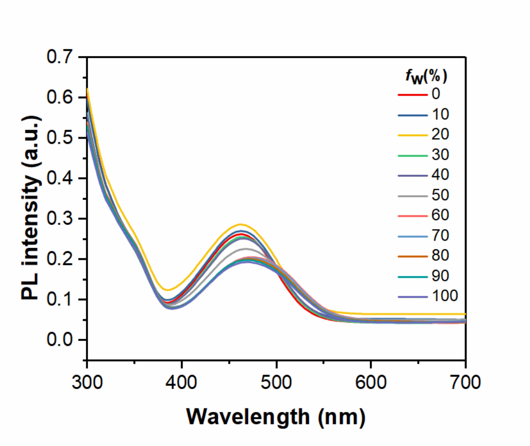

Figure S5. The absorption spectra in function of the water fraction of BY1 in DMSO.

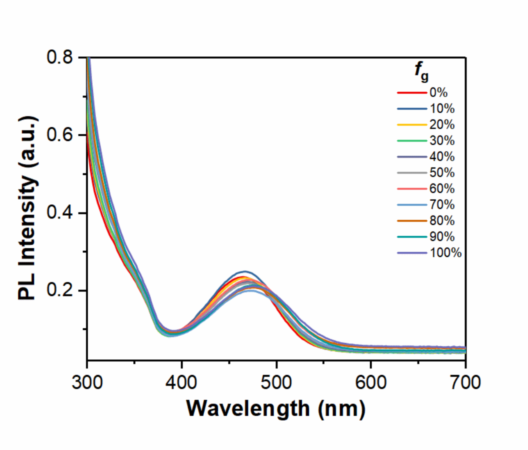

Figure S6. The absorption spectra in function of the glycerol fraction of **BY1** in EtOH.

Table S1. The photo-physical of **BY1** in different solvent.

	Solvents	λabs(nm) [a]	λem(nm) [b]	Stokes shifts(nm) [c]	$\Delta v(cm^{-1})$ [d]
BY1	DMSO	462	696	234	7277
	DMF	460	674	214	6902
	EtOH	464	656	192	6307
	TCM	476	630	154	5135
	THF	460	630	170	5866
	DCM	470	650	180	5891

[a] Absorption maxima, [b] Fluorescence emission maxima, [c] Stokes shifts, [d] $\Delta v = (vA - vF) = 1/\lambda abs - 1/\lambda em$, vA and vF are the wavenumbers (cm^{-1}) of the absorption and emission.

Table S2. Crystal data and structure refinement for **BY1**

Identification code	BY1BINGTONGZHENGJIWAN_RT_auto
Empirical formula	$C_{36}H_{27}NO_4S$
Formula weight	569.64
Temperature/K	300.75(16)
Crystal system	monoclinic

Space group	P2/c
a/Å	19.4889(14)
b/Å	8.7730(6)
c/Å	20.111(2)
α/°	90
β/°	106.593(9)
γ/°	90
Volume/Å^3	3295.3(5)
Z	4
ρ_{calc}g/cm^3	1.148
μ/mm^{-1}	0.135
F(000)	1192.0
Crystal size/mm^3	0.15 × 0.13 × 0.11
Radiation	MoKα (λ = 0.71073)
2Θ range for data collection/°	4.166 to 50
Index ranges	-23 ≤ h ≤ 22, -10 ≤ k ≤ 10, -23 ≤ l ≤ 23
Reflections collected	40231
Data/restraints/parameters	5781/99/418
Goodness-of-fit on F^2	1.073
Final R indexes [I>=2σ (I)]	R_1 = 0.0529, wR_2 = 0.1526

Final R indexes [all data]	$R_1 = 0.0715$, $wR_2 = 0.1663$
Largest diff. peak/hole / e Å^{-3}	0.32/-0.48

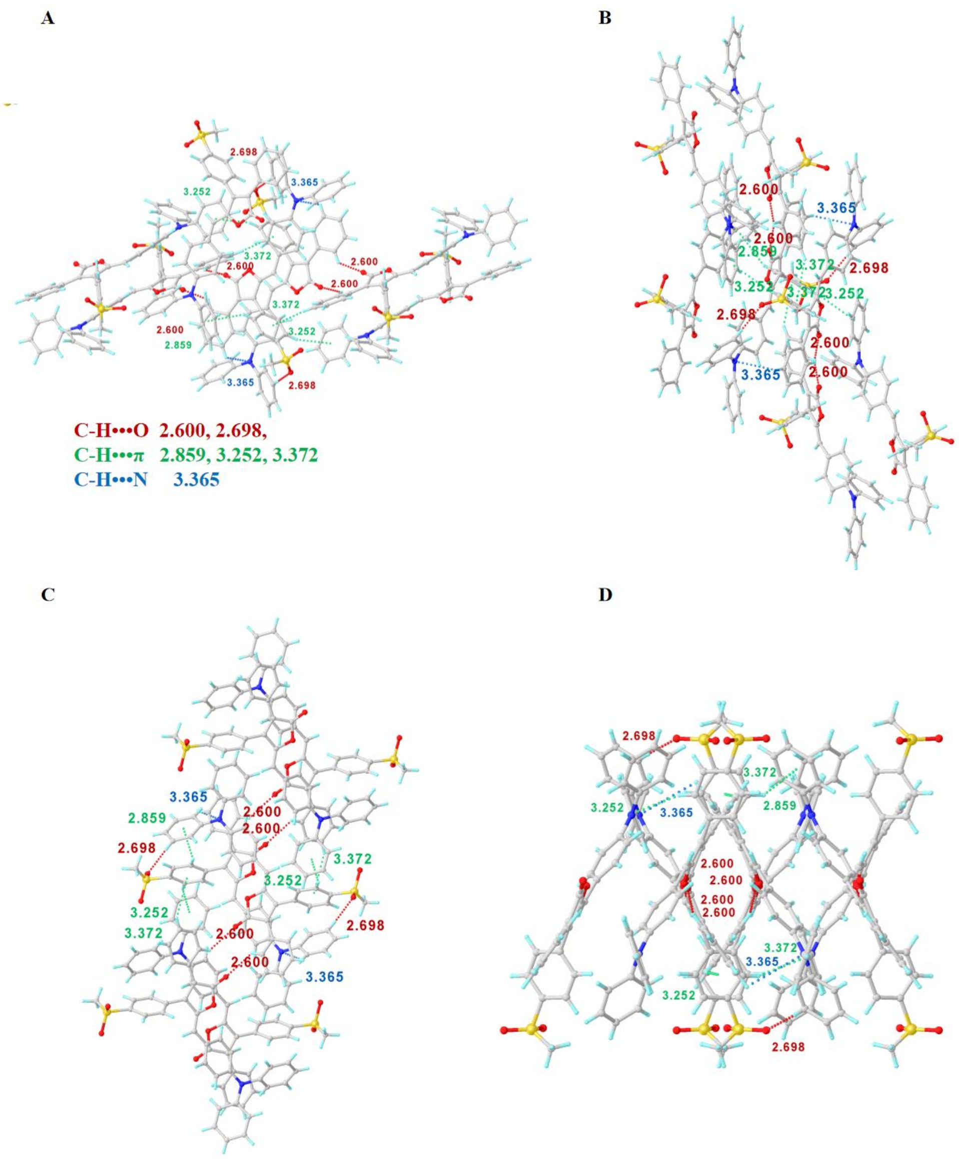

Figure S7. Packing model and interaction of **BY1**. (B),(C) side view, (D) top view

Table S3. The cell viability at different concentration.

Concentration (μM)	cell viability (%)			Mean	Standard
0.01	102.40	96.37	95.79	98.19	3.66036
0.1	91.74	99.05	103.09	97.96	5.75297

1	103.00	94.62	97.28	98.30	4.2821
10	100.21	97.85	95.90	97.99	2.15825
50	86.56	83.62	92.29	87.49	4.40918

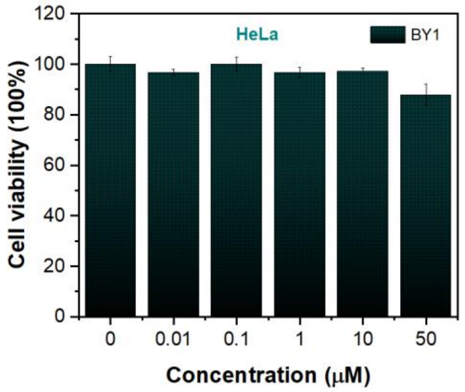

Figure S8. Cell viability values (%) estimated by CCK8 assays using Hela cells, cultured in the presence of 0.01 - 50 μM of **BY1** for 24 h at 37 °C.
